# Bisphenol F-Associated Repeated Breeding Syndrome in Cows: Epidemiological Evidence and Protective Effects of Phillyrin Against Granulosa Cell Injury

**DOI:** 10.3390/vetsci13070670

**Published:** 2026-07-09

**Authors:** Yueqi Wang, Boyang Zhang, Rui Yang, Yan Zhang, Daozhen Jiang, Yifei Mao, Bo Tang, Xueming Zhang

**Affiliations:** 1 State Key Laboratory for Diagnosis and Treatment of Severe Zoonotic Infectious Diseases, College of Veterinary Medicine, Jilin University, Changchun 130062, China or 821012510435@caas.cn (Y.W.); zby23@mails.jlu.edu.cn (B.Z.); ruiyang22@mails.jlu.edu.cn (R.Y.); z_yan22@mails.jlu.edu.cn (Y.Z.); jiangdz23@mails.jlu.edu.cn (D.J.); maoyf24@mails.jlu.edu.cn (Y.M.); tang_bo@jlu.edu.cn (B.T.); 2 State Key Laboratory of Animal Biotech Breeding, Institute of Animal Science, Chinese Academy of Agricultural Sciences (CAAS), Beijing 100193, China

**Keywords:** bisphenol F, cow, granulosa cells, ovary, phillyrin, repeat breeding syndrome

## Abstract

Repeat breeding syndrome (RBS), characterized by repeated artificial insemination failure, is associated with ovarian dysfunction in ruminants and causes economic losses. Environmental endocrine-disrupting chemicals (EDCs), such as bisphenol F (BPF), may contribute to RBS, but the relationship between BPF exposure and RBS in cows remains unclear. In this study, we conducted an epidemiological investigation and clinical examinations in 111 adult Simmental cows, including 7 cows diagnosed with RBS, from two local farms, and measured the BPF levels in the drinking water, feed, and the serum samples. We further evaluated the protective effects of phillyrin, a bioactive compound derived from *Forsythia suspensa*, against BPF-induced ovarian toxicity using cow granulosa cells (CGCs). RBS mainly occurred in young cows with two or more parities. RBS cows showed signs of potential liver dysfunction, kidney injury, anemia, and fecal abnormalities suggestive of altered gut health, together with higher serum BPF levels and lower estradiol levels. Detectable BPF contamination in the drinking water and feed suggested environmental exposure as a possible contributor to RBS in cows. In vitro, BPF induced inflammatory and apoptotic responses in CGCs by upregulating pro-inflammatory and pro-apoptotic mediators, whereas phillyrin pretreatment markedly alleviated these effects. These findings suggest that BPF exposure might be associated with RBS in cows and phillyrin could be used as a promising natural agent for mitigating BPF-related reproductive toxicity.

## Simple Summary

Repeat breeding syndrome (RBS) causes repeated artificial insemination failure and major economic losses in cattle, but its causes remain unclear. In this study, we investigated Simmental cows from two local farms and assessed whether exposure to bisphenol F (BPF), an environmental endocrine-disrupting chemical (EDC), was associated with this disorder. Cows with RBS had higher serum BPF levels and lower estradiol levels. Additionally they exhibited clinical indicators suggestive of possible liver or biliary abnormalities, along with early renal changes, anemia-related changes, and fecal abnormalities indicative of altered gut health. BPF was also detected in drinking water and feed, suggesting possible environmental exposure. In cultured cow granulosa cells, BPF triggered inflammatory and apoptotic responses. Furthermore, we found that phillyrin, a natural compound from *Forsythia suspensa*, reduced these harmful effects, indicating potential protection against BPF-related reproductive toxicity.

## 1. Introduction

Repeat breeding syndrome (RBS) has been defined as failure to conceive after three or more regularly spaced services in the absence of detectable abnormalities, which significantly impairs the reproductive efficiency of dairy cows and other ruminants, causing substantial economic losses in livestock production [1,2,3,4]. RBS is a multifactorial reproductive disorder involving abnormalities before, during, or after fertilization. It is associated with genetic or acquired defects in oocytes and spermatozoa, reproductive inflammation, endocrine dysfunction, nutritional imbalance, and management factors [1,5]. In addition, metabolic and reproductive conditions such as subclinical hypocalcemia and twin pregnancy have also been reported to impair reproductive performance, increase the likelihood of repeated inseminations, and generate additional economic losses at the herd level [6,7]. In addition, the quality of feed and drinking water is closely related to reproductive performance, and marked geographical variation in RBS prevalence has been reported across dairy farming regions [8].

Among these environmental factors, endocrine-disrupting chemicals (EDCs) have attracted increasing attention because they can interfere with hormonal signaling, ovarian function, oocyte competence, and early embryonic survival. Generally, EDCs interfere with the hormonal signaling in animal reproduction. Bisphenol F (BPF) is an EDC and a substitute for bisphenol A (BPA), which is widely used in various plastic materials [3]. Research has confirmed BPF residues in animal feed, water sources, soil, and biological samples from animals and humans [4]. Although initially regarded as a safe alternative, BPF has been found to possess endocrine-disrupting activity and potential toxicity to mouse ovarian structure and function [9]. BPA and BPF directly reduced the quality and functional competence of bovine sperm, BPA impaired early bovine embryo development and metabolism, and BPF altered estradiol secretion in bovine granulosa cells [10,11,12,13]. More importantly, the relationship between environmental BPF exposure and RBS in cows has not been systematically investigated, and it remains unclear whether BPF levels in water, feed, and serum are associated with the occurrence of RBS. Therefore, the present study aimed to evaluate BPF exposure in cow-related samples and explore its potential association with RBS.

Traditional treatments for ovarian injury, such as antibiotics and hormonal drugs, have limited efficacy against EDC-induced ovarian injury because they do not directly address oxidative stress, inflammation, mitochondrial dysfunction, or granulosa cell apoptosis [13]. In contrast, bioactive compounds from herbal medicine have gained attention due to their multi-target effects, including antioxidant, anti-inflammatory, anti-apoptotic, and endocrine-regulating activities, making them promising candidates for alleviating EDC-related ovarian damage [10,14,15,16]. *Forsythia suspensa* is an oriental medicinal herb containing bioactive compounds such as phillyrin, which has shown therapeutic potential in metabolic disorders, inflammation, infections, and cancer [15,16,17]. However, whether phillyrin can potentially be used to prevent or alleviate BPF-induced ovarian injury remains unexplored, particularly in cows. In the ovary, granulosa cells (GCs) play pivotal regulatory roles in ovarian development, and their dysfunction is associated with various ovarian disorders [18]. Thus, these cells can be used as an in vitro model for studying ovarian physiology, pathology, and reproductive toxicology, especially in large livestock such as cows [19]. Ovarian injury is difficult to diagnose under field conditions because cows often show no obvious symptoms and may only present with repeated insemination failure or RBS. This delays etiological diagnosis and timely treatment. Therefore, improving early and accurate diagnosis is important.

Based on the above-mentioned background, this study aimed to conduct an epidemiological investigation and clinical tests (blood, urine, feces) of the local RBS cows, examine the BPF levels in water, feed, serum samples, and the hormone levels in these animals. Furthermore, cow GCs (CGCs) were employed as an in vitro model to evaluate the effects of phillyrin on the BPF-induced ovarian injury in cows.

## 2. Materials and Methods

### 2.1. Materials

Phillyrin and BPF (both purity ≥ 98%) were provided by Shanghai Yuan Ye Bio-technology (Shanghai, China). Cell Counting Kit-8 (CCK-8) was supplied by Saint-Bio (Shanghai, China). The Tunel Apoptosis Detection Kit was obtained from KeyGENBioTECH (Nanjing, China). The Animal RNA Extraction Kit and lysis buffer were provided by Beyotime (Shanghai, China). The RNA Reverse Transcription Kit was obtained from TransGen Biotech (Beijing, China). Antibodies against follicle-stimulating hormone receptor (FSHR, 226665-1-AP), cyclooxygenase 2 (COX-2, 12,375–1-AP), inducible nitric oxide synthase (iNOS, 18,985–1-AP), and β-actin (66,009–1-Ig) were provided by Proteintech (Wuhan, China). Antibodies against Caspase-3 (R23727), Bax (R22708), and Bcl2 (381,702) were acquired from ZEN Bio (Chengdu, China). Horseradish peroxidase-labeled secondary antibodies were provided by Bosterbio (Wuhan, China). The BCA Protein Assay Kit was supplied by Thermo Fisher Scientific (Waltham, MA, USA). Polyvinylidene difluoride membrane was supplied by Millipore (Darmstadt, Germany). The basal medium was provided by Gibco (Oakland, CA, USA). Unless stated otherwise, other chemicals and reagents were purchased from Sigma-Aldrich (St. Louis, MO, USA).

### 2.2. Animals, Diets, and Sample Preparation

A total of 111 adult Simmental cows were enrolled from two dairy farms in Dehui and Jiutai Districts of Changchun City, Jilin Province, China, between March and August 2024. There were 63 cows from the Dehui farm (including 4 RBS cows) and 48 cows from the Jiutai farm (including 3 RBS cows). The RBS cows were diagnosed according to the standard definition and basic characteristics [1], namely failure to conceive after three or more artificial insemination at normal intervals (17–24 days) without obvious clinical disease. Seven healthy non-RBS cows from the same farms were selected as controls. These cows had regular estrous cycles, no history of repeated insemination failure, and no apparent reproductive or systemic diseases based on farm records and veterinary examinations. To reduce confounding, control cows were matched as closely as possible with RBS cows in breed, age, parity, feeding period, housing, diet, and management. All cows underwent routine veterinary examinations. Those with reproductive tract diseases, including clinical endometritis, metritis, pyometra, vaginitis, cervicitis, ovarian cysts, ovarian inactivity, or other abnormalities, were excluded. Cows with systemic diseases or abnormal conditions, such as mastitis, severe lameness, digestive or respiratory disorders, fever, metabolic diseases, or infectious diseases, were also excluded. The cows were housed in freestall barns on the two farms. Cows received a total mixed ration (Table 1) three times daily and had free access to feed and water.

Blood samples were collected by venipuncture of the jugular vein into evacuated blood collection tubes. Samples were placed on ice until arrival at the laboratory and then centrifuged at 2000× *g* for 15 min at 4 °C. Two aliquots of 2 mL of the serum were frozen at −20 °C until analysis. The perineal region of each cow was wiped with a clean paper towel to remove fecal residues, and urination was stimulated by massaging the vulva. After urination started, the first milliliters were discarded, and urine was collected into a clean beaker without preservative. Samples were immediately centrifuged at 10,000× *g* at 4 °C for 10 min, filtered through a 0.45 µm single-use syringe filter to remove debris, aliquoted, and stored at −80 °C until analysis. Fecal samples were obtained by inserting a sterile cotton-tipped swab into the rectum or from fresh feces of each cow and stored at −20 °C for subsequent analysis.

### 2.3. Isolation and Culture of CGCs

The ovaries of Simmental cows aged 2–3 years were collected from Changchun Jixing Meat Industry Co., Ltd. (Changchun, China) for the isolation and culture of CGCs. The tissues were placed in a thermos flask containing saline with penicillin-streptomycin, maintained at 37 °C, and transferred to the laboratory within 1 h. The tissues were rinsed twice with diluted bromogeramine (0.1%, *v*/*v*) and completely immersed in 75% alcohol for 10 min. Follicular fluid was aspirated using a 10 mL syringe and centrifuged at 112× *g* for 5 min. The cell pellets were washed 2–3 times with phosphate-buffered saline (PBS) containing 1% penicillin-streptomycin. The pellets were resuspended in DMEM/F12 culture medium (10% fetal bovine serum and 1% penicillin-streptomycin in DMEM/F12) and filtered through a cell strainer. The filtered cells were cultured at 37 °C with 5% CO_2_.

### 2.4. Enzyme-Linked Immunosorbent Assay (ELISA)

Feed samples were collected from the two dairy farms and mixed with 1 mL of PBS and magnetic beads. The samples were ground at 50 Hz for 20 min and then centrifuged at 12,000× *g* for 15 min at 4 °C. The supernatants were collected for total protein analysis. Serum, feed, and drinking water samples were prepared for ELISA (ELISA kits, Jiangsu Meimian Industrial Co., Ltd., Yancheng, China) according to the manufacturer’s instructions. The absorbance of each well was measured at a wavelength of 450 nm within 15 min.

### 2.5. Immunofluorescent Staining

The CGCs were fixed with 4% paraformaldehyde solution at 4 °C for 1 h. Next, the cells were permeabilized with 1% Triton X-100 for 30 min and blocked with 3% bovine serum albumin (BSA) for 1 h. After that, the primary antibody FSHR (1:200) was added, and the samples were incubated overnight at 4 °C. On the next day, the fluorescent secondary antibody (1:500) was added, and the samples were incubated for 1.5 h in the dark. The nuclei were stained with Hoechst 33342 for 5 min in the dark, and the samples were mounted with an anti-fluorescence quenching agent. Before each step mentioned above, the cells were washed three times with PBS for 5 min each. Cells stained with isotype IgG served as the negative control.

### 2.6. Cell Viability Analysis

The CCK-8 assay was applied to test whether BPF and phillyrin affected the cell viability. Briefly, BPF and phillyrin were dissolved in dimethyl sulfoxide (DMSO) to prepare stock solutions (500 mM for BPF and 100 mM for phillyrin). Stock solutions were prepared under sterile conditions, aliquoted and stored in the dark at 4 °C. Before use, they were freshly diluted with complete medium. The final DMSO concentration was kept consistent across all groups and did not exceed 0.1% (*v*/*v*). Control and vehicle groups received the same DMSO concentration without BPF or phillyrin. The CGCs were seeded and cultured in a 96-well plate. After culture for 3 h in serum-free medium, the cells were incubated with BPF at different concentrations (0.1, 0.125, 0.25, 0.5, and 1 mM, respectively) for 24 h, or treated with phillyrin (0, 10, 20, 40, 60, 80, 100, and 120 μM, respectively) for 24 h. Next, the CCK-8 solution was added (10 μL/well) and the cells were incubated for 30 min. Finally, the optical density value was measured at 450 nm.

### 2.7. Phillyrin and BPF Treatments in CGCs

According to the CCK-8 evaluation, the effects of phillyrin at 40, 60, and 80 μM against 0.5 mM BPF-induced CGC injury were further examined. Briefly, after culture for 3 h in a 96-well plate with serum-free medium, CGCs were randomly divided into 6 groups: the control group (Phillyrin−/BPF−), the BPF-treated model group (Phillyrin−/BPF+), the phillyrin-alone group (80 μM phillyrin without BPF, Phillyrin80/BPF−), the low-dose phillyrin plus BPF group (40 μM phillyrin + 0.5 mM BPF, Phillyrin40/BPF+), the medium-dose phillyrin plus BPF group (60 μM phillyrin + 0.5 mM BPF, Phillyrin60/BPF+), and the high-dose phillyrin plus BPF group (80 μM phillyrin + 0.5 mM BPF, Phillyrin80/BPF+). Cells were pretreated with or without phillyrin for 2 h, followed by incubation with or without 0.5 mM BPF for 24 h. Each group contained three independent biological replicates, and each biological replicate included three technical replicates. After treatments, the samples were used for Tunel staining, qRT-PCR, and Western blot analysis.

### 2.8. Tunel Staining

The cells were fixed with 4% paraformaldehyde for 30 min after washing with PBS for 5 min. Subsequently, the cells were incubated with PBS containing 0.3% Triton X-100 for 5 min and then washed twice with PBS. Apoptosis was detected using the Tunel Kit according to the manufacturer’s instructions. The samples were mounted with an anti-fluorescence quenching mounting medium. The Tunel-positive cells (green) were counted, and the numbers were statistically analyzed among groups.

### 2.9. Quantitative Real-Time Polymerase Chain Reaction (qRT-PCR)

Total RNA was extracted from the CGC samples and the cDNA was obtained using the RNA Reverse Transcription Kit. The primers (Table 2) were designed according to the gene sequences in the NCBI database and synthesized by Sangon Biotech (Shanghai, China). The qRT-PCR systems of 20 µL were prepared and the reactions were carried out according to the manufacturer’s instructions. The experiments were repeated three times independently, and relative gene expression was calculated according to the formula 2^−∆∆CT^ [20].

### 2.10. Western Blotting

The cell samples were lysed and centrifuged at 12,000× *g* for 15 min. The supernatant was collected, and protein concentrations were measured using a BCA Protein Assay Kit. The proteins were separated by 6–15% SDS-PAGE at 110 V for 90 min and then transferred to a polyvinylidene difluoride membrane at 75 V for 1 h. The membrane was blocked with 5% skim milk in 0.1% Tris-buffered saline-Tween (TBS-T) for 2 h, followed by overnight incubation at 4 °C with primary antibodies diluted in 5% BSA. After washing 5 times with TBS-T, the membranes were co-incubated with the corresponding horseradish peroxidase-labeled secondary antibodies (1:3000) at room temperature for 1 h. Finally, the immunoreactive bands were visualized with an enhanced chemiluminescent substrate.

### 2.11. Statistical Analysis

In the animal investigation, each cow was regarded as an independent biological replicate, and *n* represents the number of animals in each group. In the cell experiments, all assays were independently repeated three times using separately cultured CGCs as biological replicates, and each biological replicate included three technical replicates. Data were presented as mean ± SEM and analyzed using GraphPad Prism 10 (La Jolla, CA, USA). Differences between groups were analyzed by parametric one-way analysis of variance (ANOVA), and pairwise comparisons were performed using the least-significant difference test. To address concerns about multiple comparisons, we verified all significant findings using Tukey’s HSD post hoc test. All reported primary endpoint differences remained significant, confirming the robustness of our conclusions. *p* < 0.05 is considered statistically significant (* *p* < 0.05, ** *p* < 0.01, *** *p* < 0.001). All experiments were all carried out in triplicate.

For Western blot quantification, β-actin served as the internal control. Band intensities of target proteins and β-actin were measured using ImageJ 2.0. Target protein signals were normalized to the corresponding β-actin signals, and fold changes were calculated relative to the control group, which was set to 1. All experiments were performed in triplicate under identical exposure and imaging conditions. Data are presented as mean ± SEM and were analyzed using the statistical methods described above.

## 3. Results

### 3.1. Epidemiological Investigation, Clinical Tests, and Sampling Detection of BPF

**Figure 1:**
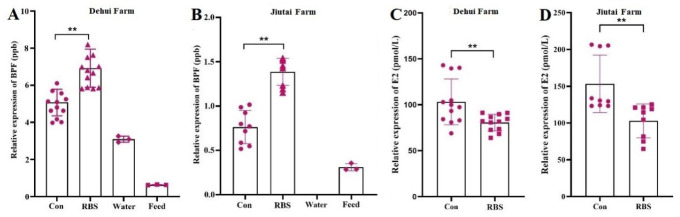
BPF and E2 levels were determined by ELISA. (**A**,**B**) BPF levels in the serum, water, and feed samples in Dehui farm (**A**) and Jiutai farm (**B**). (**C**,**D**) Serum E2 levels of the cows in Dehui farm (**C**) and Jiutai farm (**D**). Con = control, all values are shown as mean ± SEM, ** *p* < 0.01.

Epidemiological investigation showed that 7 of 111 Simmental cows had RBS, with an overall occurrence of 6.31%. The occurrence was 6.35% (4/63) in the Dehui farm and 6.25% (3/48) in the Jiutai farm. The RBS cows were 2–4 years old, with parity ≥ 2, and some showed pica-like symptoms. Blood, urine, and fecal routine tests were conducted in RBS and control cows (Table 3, Table 4 and Table 5). RBS cows showed lower red blood cell counts and hemoglobin levels, suggesting possible anemia. Some cows had increased urinary protein and microalbumin, indicating possible early kidney changes. Elevated bilirubin-related indicators suggested possible liver or biliary abnormalities. Fecal tests showed white blood cells, epithelial cells, and Vibrio in some RBS cows, suggesting possible intestinal inflammation or infection. These findings were considered preliminary indicators rather than definitive diagnoses. Furthermore, the serum, feed, and drinking water samples were analyzed using ELISA. The results demonstrated that the water and feed from the Dehui farm contained detectable BPF (Figure 1A), while BPF was undetectable in the drinking water from the Jiutai farm (Figure 1B). In both farms, the serum BPF levels were significantly higher and the estradiol (E2) levels were remarkably lower in the RBS cows than those in the control (Con) cows (Figure 1B,C). These results suggest that higher BPF exposure may be associated with reduced E2 levels and ovarian dysfunction in RBS cows, although no direct causal relationship can be confirmed.

### 3.2. BPF Induces Inflammatory and Apoptotic Responses in CGCs

**Figure 2:**
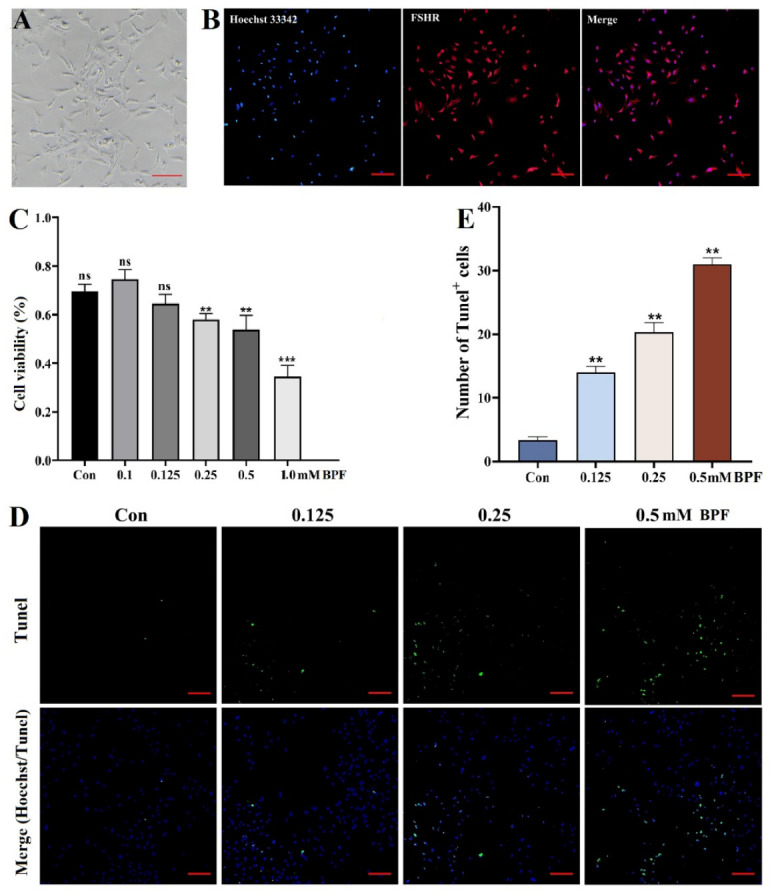
Identification of CGCs and Tunel staining of the BPF-treated CGCs. (**A**) Morphology of the CGCs cultured for 24 h. (**B**) Immunostaining of follicle-stimulating hormone receptor (FSHR). (**C**) CCK-8 assay detects the effect of BPF on CGC viability. (**D**) Representative fluorescent staining images of Tunel staining in the CGCs treated with BPF at 0, 0.125, 0.25, and 0.5 mM, respectively. (**E**) Statistical analysis of the Tunel^+^ cell numbers. Scale bar = 100 μm in (**A**) and 200 μm in (**B**) and (**D**); ns = not significant, ** *p* < 0.01, and *** *p* < 0.001.

To examine the response of the cow ovary to BPF exposure, the CGCs were employed as an in vitro model. The primary CGCs showed spindle-shaped or polygonal appearances, forming extended structures resembling fibroblasts (Figure 2A). Immunostaining of the GC marker FSHR was performed, confirming that these cells were granulosa cells with a purity > 95% (Figure 2B). Next, the CCK-8 assay indicated that mild cell damage occurred after 0.125 mM BPF treatment, severe damage started after 0.25 mM BPF incubation, and the cell viability decreased dramatically after 1.0 mM BPF treatment. Thus, BPF treatment at concentrations of 0.125, 0.25, and 0.5 mM were selected for subsequent experiments (Figure 2C). Furthermore, Tunel staining revealed that BPF stimulation induced apoptosis, with a dose-dependent increase in apoptotic cells in the BPF-treated groups (Figure 2D,E).

**Figure 3:**
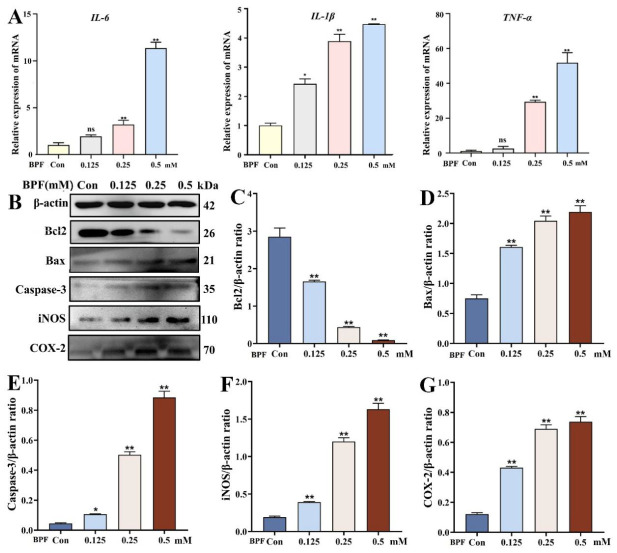
BPF induces inflammatory and apoptotic responses in the CGCs. (**A**) qRT-PCR analysis showed that BPF treatment upregulated the mRNA expressions of the inflammatory gene *IL-6*, *IL-1β* and *TNF-α* in a dose-dependent manner in the CGCs. (**B**) Western blot analysis of the anti-apoptosis protein Bcl2, apoptotic protein Bax and Caspase-3, and the pro-inflammatory mediator iNOS and COX-2 in BPF-treated CGCs, with β-actin as the reference (the original Western-blotting pictures can be found in File S1). (**C**–**G**) Bar graphs represent the quantitative analysis of the corresponding protein bands in (**B**); ns = not significant, * *p* < 0.05, and ** *p* < 0.01.

Subsequently, qRT-PCR was performed to evaluate the impact of BPF exposure on the inflammatory responses in the CGCs. As shown in Figure 3A, BPF significantly upregulated the mRNA expression levels of the pro-inflammatory gene interleukin-6 (*IL-6*), *IL-1β*, and tumor necrosis factor-α (*TNF-α*). Western blot analysis further showed that BPF-treated CGCs exhibited significantly elevated protein levels of immunoregulatory factors (iNOS and COX-2) and apoptotic molecules (Caspase-3 and Bax), along with decreased expression of the anti-apoptotic protein Bcl2 (Figure 3B–G). Together, these data demonstrate that BPF induces inflammatory responses and apoptosis in the CGCs in a dose-dependent manner, with escalating effects at higher concentrations.

### 3.3. Phillyrin Pretreatment Alleviates BPF-Induced Inflammatory-Apoptotic Response in CGCs

**Figure 4:**
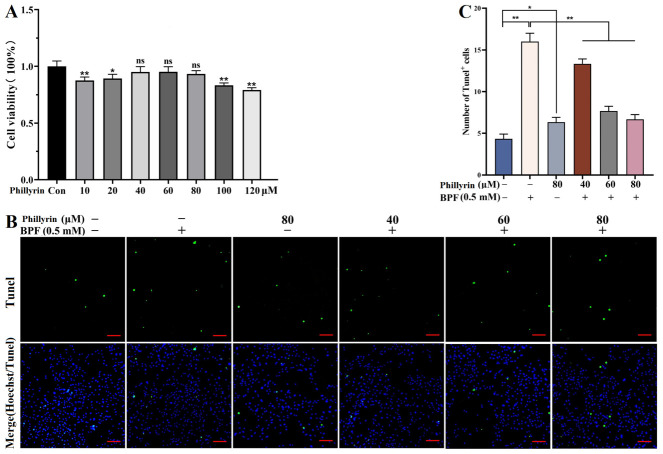
Effects of phillyrin pretreatment on BPF-induced apoptosis in CGCs. (**A**) CCK-8 assay showed the effects of phillyrin at different concentrations on CGC viability; accordingly concentrations of 40, 60, and 80 μM were selected for subsequent tests. (**B**) Tunel staining in different groups, including Phillyrin−/BPF−, Phillyrin−/BPF+, Phillyrin80/BPF−, Phillyrin40/BPF+, Phillyrin60/BPF+, and Phillyrin80/BPF+ (scale bar = 200 μm). (**C**) Bar graph of Tunel^+^ cell numbers for the corresponding groups in (**B**); ns = not significant, * *p* < 0.05, and ** *p* < 0.01.

Given the broad pharmacological effects of phillyrin, we examined its protective effects against BPF-induced inflammatory and apoptotic responses in the CGCs. Firstly, the CCK-8 assay was performed to evaluate the impact of phillyrin on the CGC viability. Compared with the Con group, no significant effect was observed when these cells were incubated with phillyrin at concentrations of 40, 60, and 80 μM respectively (Figure 4A). Thus, these concentrations were selected for the subsequent tests. Furthermore, Tunel staining confirmed that, compared with the Phillyrin−/BPF+ group, phillyrin treatment effectively suppressed BPF (0.5 mM)-induced apoptosis and markedly decreased the apoptotic rates in the Phillyrin40/BPF+, Phillyrin60/BPF+, and Phillyrin80/BPF+ groups, which were close to the levels in the PHI−/BPF− and PHI80/BPF− groups (Figure 4B,C).

**Figure 5:**
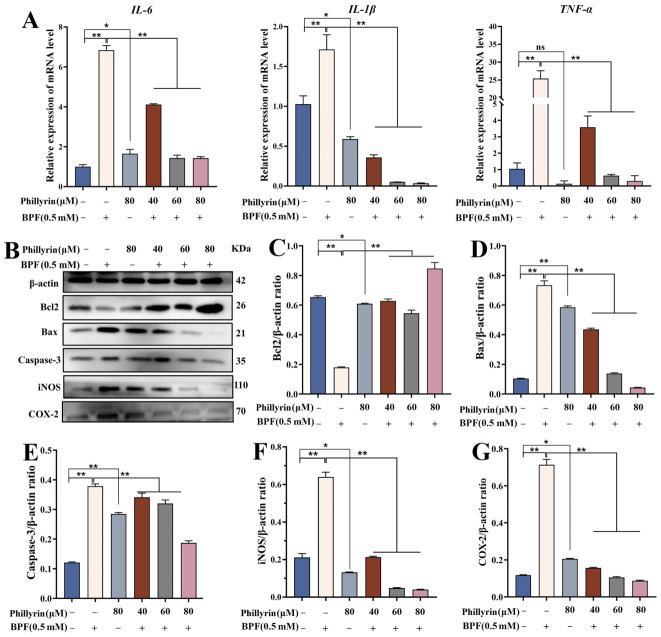
Phillyrin pretreatment alleviates BPF-induced inflammatory-apoptotic responses in the CGCs. (**A**) The mRNA levels of IL-6, IL-1β, and TNF-α in Phillyrin/BPF treated groups were detected by qRT-PCR. (**B**) Western blot analysis of the apoptosis-related proteins (Bcl2, Bax, and Caspase-3) and pro-inflammatory mediators in Phillyrin/BPF treated groups, using β-actin as the reference(the original Western-blotting pictures can be found in File S1). (**C**–**G**) Bar graphs represent quantitative analysis of the corresponding protein bands in (**B**); ns = not significant, * *p* < 0.05, and ** *p* < 0.01.

Subsequently, qRT-PCR analysis showed that, compared with the Phillyrin−/BPF+ group, phillyrin pretreatment significantly reduced the mRNA expression levels of *IL-6*, *IL-1β*, and *TNF-α* in the Phillyrin40/BPF+, Phillyrin60/BPF+, and Phillyrin80/BPF+ groups (Figure 5A). Consistently, Western blot analysis showed that phillyrin pretreatment remarkably restored Bcl2 levels and downregulated Bax, Caspase-3, and the pro-inflammatory mediator iNOS and COX-2 (Figure 5B–G). Collectively, these results demonstrate that phillyrin effectively alleviates BPF-induced inflammatory responses and apoptosis in the CGCs by suppressing the pro-inflammatory mediators and modulating the apoptosis-related molecular balance.

## 4. Discussion

RBS is a reproductive disorder that reduces dairy cow fertility. In this study, we investigated whether environmental BPF exposure is associated with RBS through epidemiological, clinical, and cellular analyses. Field surveys of 111 Simmental cows indicated that RBS was more common in younger cows with multiple parities. Clinical examinations revealed physiological abnormalities in RBS cows, while BPF was detected in feed, drinking water, and serum, suggesting potential exposure-related reproductive effects. In bovine granulosa cells, BPF reduced cell viability and induced oxidative stress, inflammation, and apoptosis, whereas phillyrin alleviated these injuries. These findings suggest a potential association between BPF-related ovarian dysfunction and RBS, and also indicate that phillyrin may have protective value against BPF-induced granulosa cell injury.

In the blood tests, reduced neutrophils in RBS cows may indicate altered innate immunity. As an EDC, BPF can affect immune and hematopoietic functions. Studies in poultry and aquatic animals have shown that bisphenol exposure alters immune organs and blood indices, including WBCs and neutrophils [21,22,23]. Monocytes are important for clearing pathogens and cellular debris. A decrease in monocytes may suggest suppressed hematopoietic function or certain chronic diseases. It is often accompanied by a decrease in neutrophils and may be caused by viremia [21], sepsis, or immunosuppressive diseases. An increase in the percentage of lymphocytes usually corresponds to a decrease in neutrophils, which may indicate that the cow is in the recovery phase of an infection or has certain chronic diseases [22]. A low number of red blood cell count and a low hemoglobin concentration suggest that the cow may have anemia. The causes of anemia may include malnutrition (e.g., iron, vitamin B12, or folic acid deficiency), chronic blood loss, parasitic infection, or chronic diseases.

In the urine tests, a positive urine bilirubin result usually indicates abnormal liver function or biliary obstruction. Creatinine is an important metabolic product excreted by the kidneys, and an increase in creatinine may indicate impaired renal function, which may be related to the decreased glomerular filtration rate. Increased creatinine may be caused by acute or chronic renal insufficiency, glomerulonephritis, pyelonephritis, etc. The urine protein-to-creatinine ratio is an important indicator for evaluating kidney damage [23]. If the levels of urine protein and creatinine are high, but the ratio between them is still within the normal range, it suggests that the kidney damage may be in an early stage. Comprehensive examination of the renal function indicators (e.g., serum creatinine and blood urea nitrogen) and renal ultrasound is needed to determine whether kidney damage or a metabolic disorder is present.

The detection of Vibrio, white blood cells, epithelial cells, and yeast in feces from RBS cows indicated fecal abnormalities suggestive of altered gut health [24]. These abnormalities may be associated with BPF exposure, which has been reported to alter gut microbiota, impair intestinal barrier function, and promote inflammatory responses. Therefore, the fecal findings may reflect BPF-related intestinal dysbiosis and mucosal inflammation. However, this interpretation remains speculative because gut microbiota sequencing and intestinal barrier assessments were not performed in this study [25,26,27]. Collectively, the RBS cows may have intestinal inflammation, infection, or flora imbalance, and further evaluation is needed to determine whether bacterial enteritis or chronic enteritis is present.

ELISA analysis of the serum, feed, and drinking water clearly indicated that the serum from RBS cows and their feed and water samples (except drinking water from the Jiutai farm) contained detectable BPF. Since BPF is one of the EDCs widely used in various plastic materials, we logically compared the E2 levels between the RBS cows and the control animals. As expected, the E2 levels in the RBS cows were significantly decreased. Combined with the above clinical tests, these data together suggest that environmental BPF exposure may enter cow serum through water and feed and may be associated with endocrine disorder and RBS-related clinical findings. Previously, the effects of BPF on liver and kidney function and the metabolism have been reported [25,26]. Here, we focused on its influence on the ovary because of the disturbed E2 levels in the RBS cows.

In the present study, serum E2 levels were decreased in RBS cows, suggesting that impaired ovarian steroidogenesis may be involved in repeated breeding. This interpretation is supported by previous studies showing that repeat-breeder cows exhibit reduced oocyte competence and impaired blastocyst development, and that part of the RBS etiology may originate during early folliculogenesis [27,28]. GCs are the major ovarian cells responsible for FSH-responsive E2 synthesis and are essential for maintaining the follicular microenvironment, oocyte competence, and embryo quality [29,30]. Importantly, BPA and BPF have been reported to induce oxidative stress and apoptosis in bovine GCs, while BPA can impair early bovine embryo development and metabolism [12,31,32]. Therefore, the decreased serum E2 observed in RBS cows may be linked to GC dysfunction, supporting the use of CGCs to evaluate BPF-induced ovarian cellular injury. Accordingly, we employed CGCs as the in vitro model to examine the effects of the environmental pollutant BPF on the cow ovary.

In this study, the primary CGCs were isolated and characterized by FSHR immunostaining. The cytotoxicity of BPF was evaluated by CCK-8 assay, and further confirmed by Tunel staining. These results clearly indicate that BPF indeed is harmful to the CGCs and can induce CGC apoptosis in a dose-dependent manner. Subsequent qRT-PCR and Western blot analyses further demonstrated that BPF induced inflammatory and apoptotic responses by upregulating the expression of inflammatory cytokines and apoptotic proteins while downregulating the anti-apoptotic protein Bcl2, all in a dose-dependent manner. Inflammation and apoptosis are typical responses leading to tissue damage. Previous studies have shown that BPF and BPA induce apoptosis and oxidative stress in mouse Leydig cells [33] and human GCs [34]. BPF can also disrupt mouse intestinal homeostasis by regulating inflammatory cytokine levels [35]. The BPF concentration used in the in vitro CGC experiments should be regarded as a toxicological challenge concentration rather than a direct simulation of environmental exposure. Although higher than typical environmental levels, it was selected based on preliminary CCK-8 screening to establish a stable and reproducible CGC injury model. Previous studies have demonstrated BPF toxicity in multiple systems, including impaired neural stem cell function in vitro [36], reproductive dysfunction in rats [37], developmental neurotoxicity associated with EDC exposure [38], effects on human osteoblasts [39], and systemic and intestinal toxicity following oral exposure in rats [40]. These findings indicate that BPF can induce toxicity in different tissues depending on dose and exposure duration. However, the concentration used in cultured CGCs cannot be directly extrapolated to serum or ovarian tissue levels in cows. Therefore, this model primarily serves as a short-term mechanistic hazard-identification system rather than a chronic low-dose exposure model. Previous studies have shown that BPA and BPF reduce bovine granulosa cell viability, increase oxidative stress, and induce mitochondrial apoptosis [31,32]. Consistent with these findings, BPF-induced CGC injury may begin with excessive reactive oxygen species (ROS) accumulation and weakened antioxidant defenses, leading to NF-κB activation and increased expression of inflammatory mediators such as TNF-α, IL-6, IL-1β, COX-2, and iNOS [41]. Sustained inflammation may further disrupt mitochondrial function, resulting in BAX/BCL-2 imbalance, cytochrome c release, and caspase-dependent apoptosis [42]. Studies on BPF toxicity also suggest the involvement of the Nrf2/NF-κB axis, PI3K-AKT signaling, p53/BCL-2 regulation, and estrogen receptor-related pathways [43,44]. Therefore, BPF may act through interconnected oxidative stress, inflammatory, and apoptotic pathways in CGCs. The protective effect of phillyrin may be related to its ability to restore redox balance and suppress inflammation and apoptosis, although the underlying molecular targets require further investigation.

Given the multiple pharmacological activities of phillyrin, the main effective compound phillyrin from the herbal medicine *Forsythia suspensa*, we next explored its protective effect against the BPF-induced inflammation-apoptosis responses based on the CGC model. Phillyrin concentrations were selected based on preliminary CCK-8 and protective-effect screening assays. Concentrations of 40, 60, and 80 μM showed no obvious cytotoxicity and displayed protective effects against BPF-induced injury. These doses were therefore used to assess the ability of phillyrin to alleviate oxidative stress, inflammation, and apoptosis. The Phillyrin80/BPF− group was included to confirm the safety of the highest concentration tested. Our results demonstrate that phillyrin pretreatment dose-dependently inhibited the BPF-induced apoptosis and inflammatory responses in the CGCs by decreasing the expression of pro-inflammatory factors and pro-apoptotic proteins and restoring the anti-apoptotic protein level. Previous studies have demonstrated that phillyrin exerts protective effects in multiple injury models, although its primary mechanism varies with the pathological context. In Lipopolysaccharides-induced acute lung injury, phillyrin mainly suppresses inflammation through inhibition of MAPK and NF-κB signaling [45]. In H_2_O_2_-induced oxidative damage models, it acts predominantly as an antioxidant and anti-apoptotic agent by enhancing cellular antioxidant defenses and reducing mitochondrial apoptosis [46]. In high glucose-stimulated glomerular mesangial cells, phillyrin alleviates oxidative stress and inflammation partly via regulation of the PI3K/Akt pathway [43]. Unlike these models, the present study investigated BPF-induced toxicity in bovine ovarian granulosa cells. Our findings indicate that phillyrin protects CGCs by attenuating the oxidative stress–inflammation–apoptosis cascade triggered by BPF. Phillyrin improved cell viability, reduced oxidative and inflammatory damage, and inhibited apoptosis, suggesting a protective role through maintenance of redox balance, suppression of inflammatory signaling, and prevention of mitochondrial apoptotic pathways. These results extend the pharmacological application of phillyrin to EDC-induced ovarian granulosa cell injury in cattle, a model highly relevant to reproductive toxicity.

Several limitations should be acknowledged. The field study included a relatively small number of RBS cows from only two farms, and residual confounding factors could not be completely excluded. In addition, the cross-sectional design limits causal inference between BPF exposure and RBS. BPF concentrations were measured by ELISA, and environmental exposure sources may not have been fully characterized. Although the blood, urine, and fecal abnormalities were observed, they were not validated by targeted analyses such as microbiota profiling, intestinal barrier assessment, or tissue pathology, making the proposed associations between BPF exposure and systemic dysfunction largely speculative. Furthermore, the in vitro CGC model cannot fully reproduce the ovarian microenvironment, and the signaling pathways underlying BPF toxicity and phillyrin protection were not directly confirmed. The efficacy and safety of phillyrin in cows also remain unclear. Therefore, larger longitudinal studies and further in vivo mechanistic investigations are needed.

## 5. Conclusions

**Figure 6:**
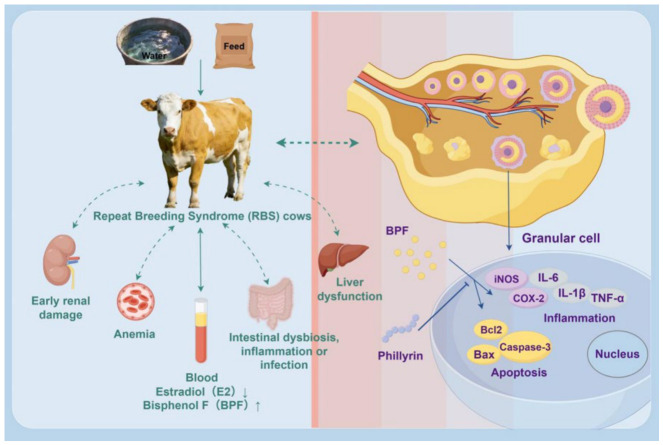
Schematic illustration of the experiments (By Figdraw 2.0). Blood, urine, and fecal tests indicate the RBS cows had potential liver/kidney dysfunction, anemia, and fecal abnormalities suggestive of altered gut health. BPF exposure may enter the blood, decrease E2 secretion, and contribute to RBS. The ovarian toxicity of BPF was confirmed in the CGC model, and can be countered with phillyrin, an active compound from the herbal medicine *Forsythia suspensa*.

This study combined field investigation, clinical examination, environmental BPF detection, and in vitro CGC experiments to explore the potential relationship between BPF exposure and RBS in cows. The epidemiological results showed that RBS was observed in 7 of 111 cows and was mainly found in relatively young cows after two or more parities. These cows exhibited decreased serum E2 levels together with clinical indicators suggestive of possible hepatic, renal, hematological, and fecal abnormalities. BPF was detected in feed, drinking water, and serum samples, suggesting that environmental BPF exposure may be one of the possible contributors associated with the occurrence of RBS. However, because the field investigation was observational, these findings indicate an association rather than direct causality. In vitro, BPF induced inflammatory and apoptotic responses in the CGCs, while phillyrin alleviated BPF-induced cellular injury by suppressing inflammation and apoptosis (Figure 6). These results provide preliminary evidence that BPF may impair bovine ovarian granulosa cell function and phillyrin may serve as a candidate protective agent against BPF-induced granulosa cell injury in vitro. Further longitudinal studies, low-dose chronic exposure models, and in vivo validation are required to clarify the potential contribution of BPF in RBS and to evaluate the practical application of phillyrin in cattle reproductive health.

## Figures and Tables

**Table 1:** Diet formulation.

Items	(%)	Nutrient Composition	(% of Dry Matter)
Corn silage	40.0	NEL (net energy for lactation, MJ/kg)	6.7
Corn	35.0	CP (crude protein)	15.2
Wheat bran	8.0	NDF (neutral detergent fiber)	33.5
Soybean meal	5.0	ADF (acid detergent fiber)	17.2
Sunflower seed	8.0	NFC (non-fiber carbohydrate)	40.4
NaCl	1.0	Ca	0.7
Premix ^#^	1.8	P	0.5
NaHCO_3_	1.2		
Total	100.0		

Note: ^#^ The premix provided the following per kg of premix: vitamin A (200,000 IU), vitamin D (70,000 IU), vitamin E (1000 IU), Fe (2000 mg), Cu (600 mg), Zn (2400 mg), Mn (1300 mg), I (6 mg), and Co (7 mg).

**Table 2:** Sequences of the primers used for qRT-PCR.

Gene	Primer Sequence
*IL-6*	F:5′-AACGAGTGGGTAAAGAACGC-3′ R:5′-CTGACCAGAGGAGGGAATGC-3′
*IL-1β*	F:5′-CTGAACCCATCAACGAAA-3′ R:5′-ATGACCGACACCACCTGC-3′
*TNF-α*	F:5′-TCGTATGCCAATGCCCTCA-3′ R:5′-GATGAGGTAAAGCCCGTCAGT-3′
*β-actin*	F:5′-GTCAGGTCATCACTATCGGCAAT-3′ R:5′-AGAGGTCTTTACGGATGTCAACGT-3′

**Table 3:** Blood routine tests (Complete blood count, CBC).

Index	RBS	Con	Reference Range
WBC (10^9^/L)	4.58 ± 1.08	4.93 ± 0.29	4.00–12.00
Neu# (10^9^/L)	0.53 ± 0.19 *	0.78 ± 0.14	0.60–6.94
Lym# (10^9^/L)	3.97 ± 0.93	4.86 ± 0.87	1.50–10.62
Mon# (10^9^/L)	0.05 ± 0.02 *	0.11 ± 0.05	0.30–2.17
Eos# (10^9^/L)	0.03 ± 0.02 **	0.11 ± 0.05	0.00–2.00
Bas# (10^9^/L)	0	0	0.00–0.20
Neu% (%)	11.3 ± 2.47 **	15.03 ± 0.49	15.0–65.0
Lym% (%)	86.8 ± 2.65 **	78.8 ± 3.27	40.0–75.0
Mon% (%)	1.1 ± 0.16 **	1.83 ± 0.25	2.0–10.0
Eos% (%)	0.73 ± 0.52 **	1.5 ± 0.36	0.0–20.0
Bas% (%)	0.07 ± 0.05	0.13 ± 0.06	0.0–2.0
RBC (10^12^/L)	4.59 ± 0.76 **	7.01 ± 0.71	5.00–10.10
HCT (%)	22.27 ± 3.51 **	29.97 ± 1.94	24.0–46.0
MCV (fL)	48.7 ± 0.62	49.23 ± 3.21	40.0–60.0
MCH (pg)	17.07 ± 1.96	15.53 ± 0.87	11.0–19.0
MCHC (g/L)	350 ± 35.7	332.6 ± 17.78	300–370
RDW-CV	25.4 ± 1.26	21.47 ± 4.66	14.0–19.0
RDW-SD	54.6 ± 3.69	52.53 ± 1.4	0.1–99.9
PLT (10^9^/L)	650.67 ± 171.87 **	306.73 ± 58.18	120–820
MPV (fL)	6.53 ± 0.71	5.83 ± 0.63	3.8–8.8
PDW (fL)	12.77 ± 1.67 **	9.87 ± 1.47	0.1–30.0
PCT (%)	0.44 ± 0.16	0.25 ± 0.13	0.010–9.990

Note: WBC = White Blood Cell count, Neu# = Neutrophil count, Lym# = Lymphocyte count, Mon# = Monocyte count, Eos# = Eosinophil count, Bas# = Basophil count, Neu% = Neutrophil percentage, Lym% = Lymphocyte percentage, Mon% = Monocyte percentage, Eos% = Eosinophil percentage, Bas% = Basophil percentage, RBC = Red Blood Cell count, HCT = Hematocrit, MCV = Mean Corpuscular Volume, MCH = Mean Corpuscular Hemoglobin, MCHC = Mean Corpuscular Hemoglobin Concentration, RDW-CV = Red Cell Distribution Width—Coefficient of Variation, RDW-SD = Red Cell Distribution Width—Standard Deviation, PLT = Platelet count, MPV = Mean Platelet Volume, PDW = Platelet Distribution Width, PCT = Plateletcrit, *n* = 7. * *p* < 0.05 and ** *p* < 0.01 (RBS vs. Con).

**Table 4:** Urine routine tests.

Index	RBS	Con
LEU (Cell/μL)	0	0
KET (mg/dL)	0	0
NIT	−	−
URO	Normal	Normal
BIL (mg/dL)	0.5 *2 0 *5	0
GLU (mg/dL)	0	0
PRO (mg/dL)	<15 *4 15 *2 30 *1	<15
USG	1.02 ± 0.01	1.01 ± 0.01
pH	6.79 ± 0.59	6.42 ± 0.89
BLD (Cell/μL)	0	1.67 ± 3.73
ASC (mg/dL)	0	4.17 ± 9.32
MA (mg/dL)	<2.5 *6 ≥2.5 *1	<2.5
Ca (mg/dL)	20 *4 10 *2 ≤4 *1	20 *3 10 *3 ≤4 *1
CR (mg/dL)	≥300 *4 200 *2 100 *1	≥300 *4 200 *1 100 *1 ≤10 *1
PRO/CR	<0.2	<0.2

Note: “−” indicates negative; “*” indicates the number of cases. LEU = Leukocyte, KET = Ketone, NIT = Nitrite, URO = Urobilinogen, BIL = Bilirubin, GLU = Glucose, PRO = Protein, USG = Urine Specific Gravity, pH = Potential of Hydrogen, BLD = Blood, ASC = Ascorbic Acid, MA = Microalbumin, Ca = Calcium, CR = Creatinine, PRO/CR = Protein/Creatinine Ratio, *n* = 7.

**Table 5:** Fecal routine examinations.

Index	RBS	Con
Occult Blood	−	0
Yeast	− *3 + *3 ++ *1	0
Epithelial Cells	− *4 + *3	−
White Blood Cells (WBCs)	− *6 ++ *1	Normal
Vibrio	− *6 + *1	0
Parasites	−	0

Note: “−” indicates negative; “+” indicates positive; “++” indicates strongly positive; “*” denotes case number, *n* = 7.

## Data Availability

The original contributions presented in this study are included in the article/Supplementary Material. Further inquiries can be directed to the corresponding author.

## Supplementary Materials

The following supporting information can be downloaded at: https://www.mdpi.com/article/10.3390/vetsci13070670/s1, File S1: Original images of Western-blotting.

## Abbreviations

BPA = bisphenol A; BPF = bisphenol F; CCK-8 = Cell Counting Kit-8; CGCs = cow granulosa cells; COX-2 = cyclooxygenase 2; Con = control; EDCs = endocrine-disrupting chemicals; ELISA = Enzyme-Linked Immunosorbent Assay; E2 = estradiol; FSHR = follicle-stimulating hormone receptor; IL-6 = interleukin-6; IL-1β = interleukin-1β; iNOS = inducible nitric oxide synthase; PBS = phosphate-buffered saline; qRT-PCR = quantitative real-time polymerase chain reaction; RBS = repeat breeding syndrome; TBS-T = Tris-buffered saline-Tween; TNF-α = tumor necrosis factor-α.
