# Supplementary material for: Bisphenol F-Associated Repeated Breeding Syndrome in Cows: Epidemiological Evidence and Protective Effects of Phillyrin Against Granulosa Cell Injury
Source: Vet Sci. 2026 Jul 9;13(7):670. doi: 10.3390/vetsci13070670 (PMC13431484; doi:10.3390/vetsci13070670)

Fig3 β-actin

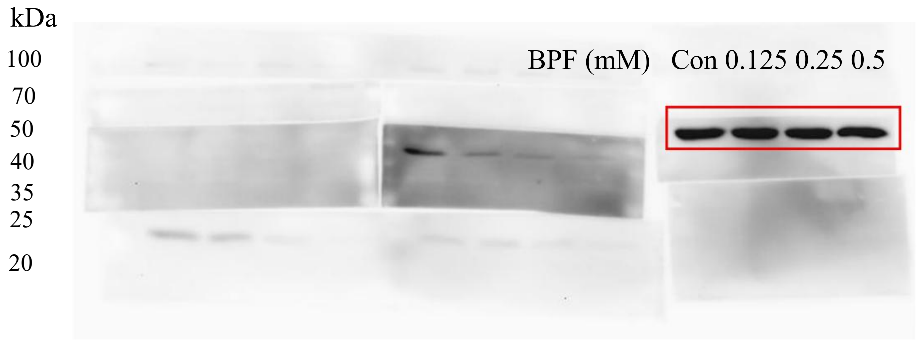

Fig3 Bcl2

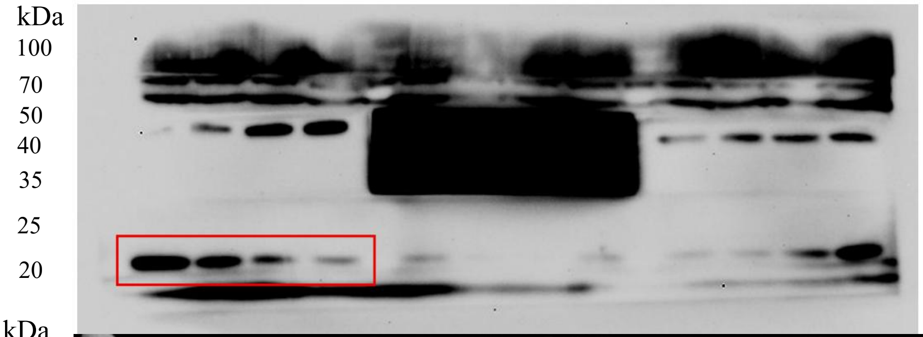

Fig3 Bax

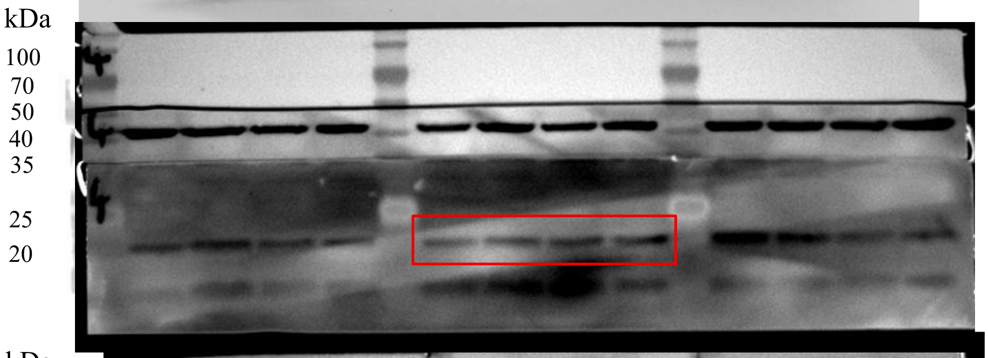

Fig3 Caspase3

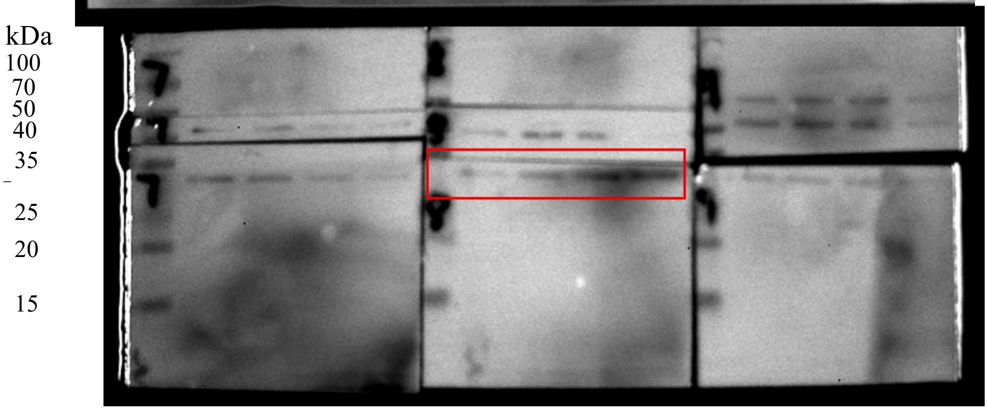

Fig3 iNOS

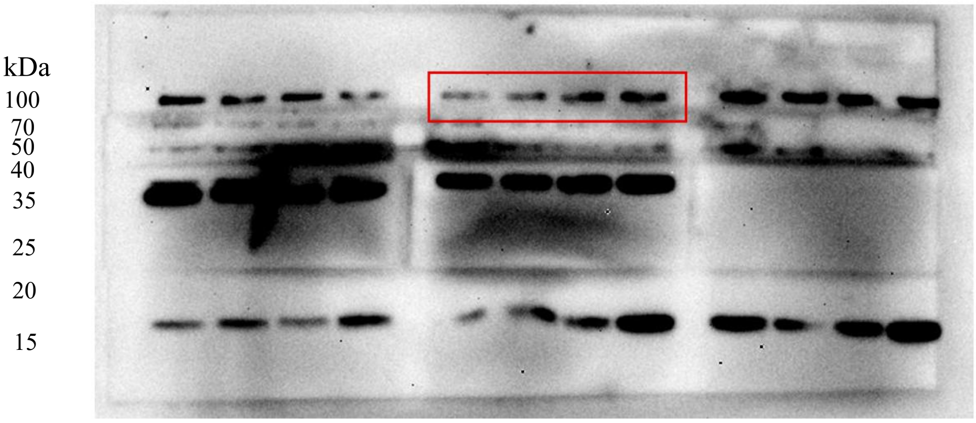

Fig3 COX-2

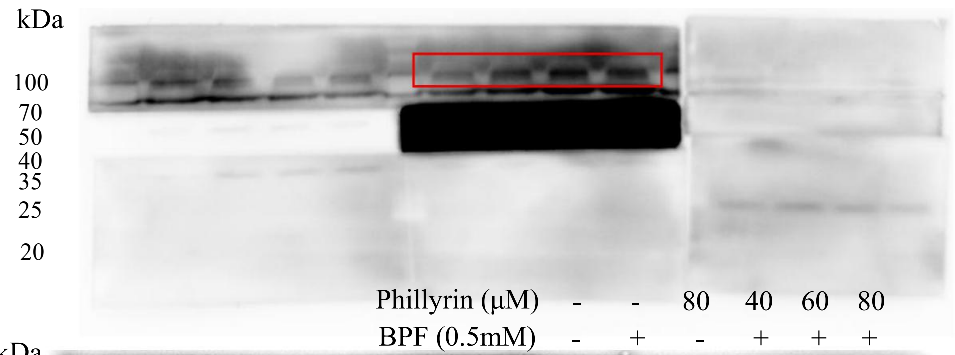

Fig5 β-actin

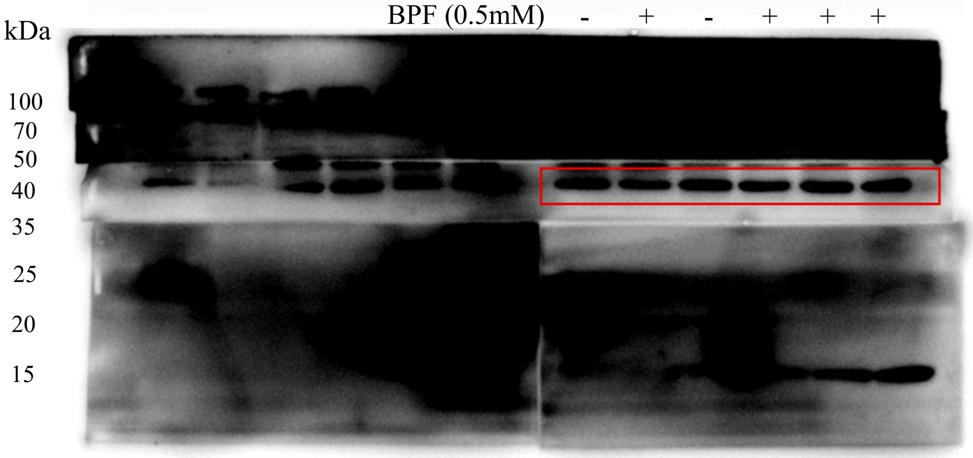

Fig5 Bcl2

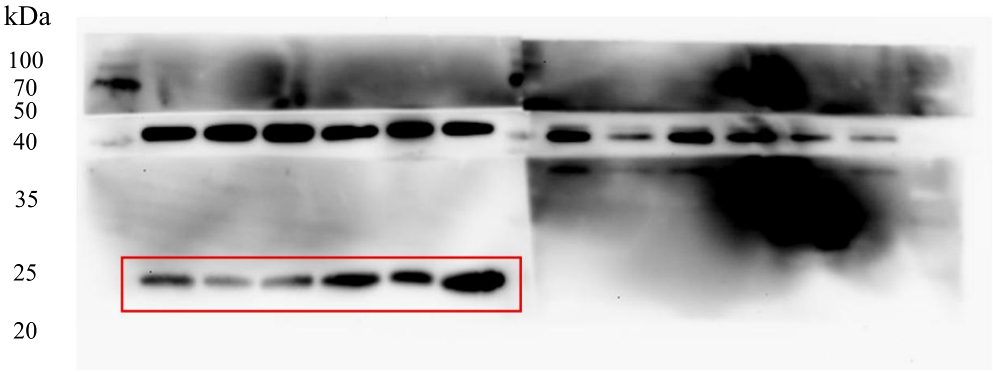

Fig5 Bax

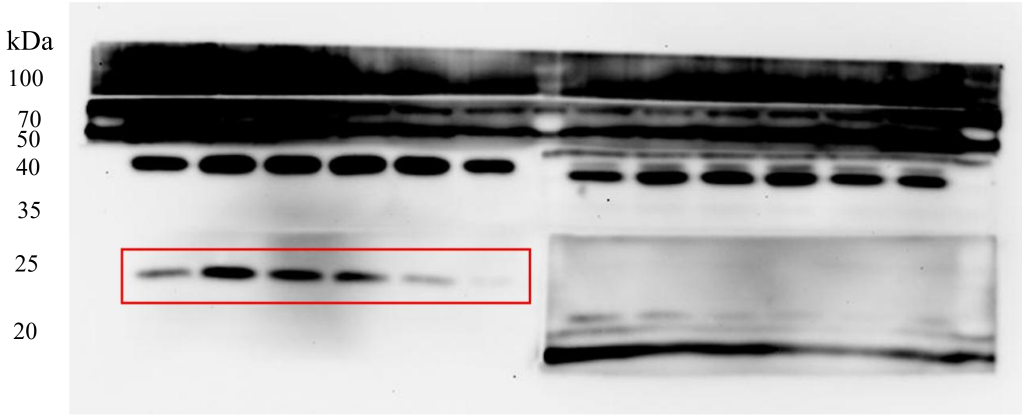

Fig5 Caspase3

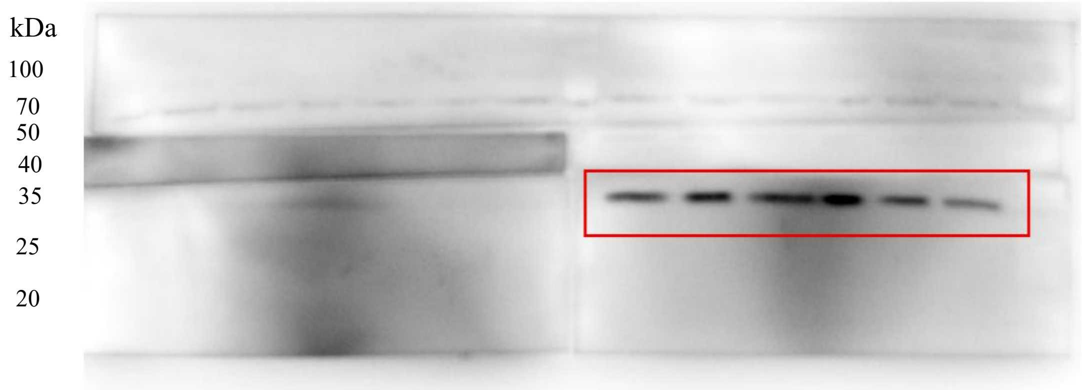

Fig5 iNOS

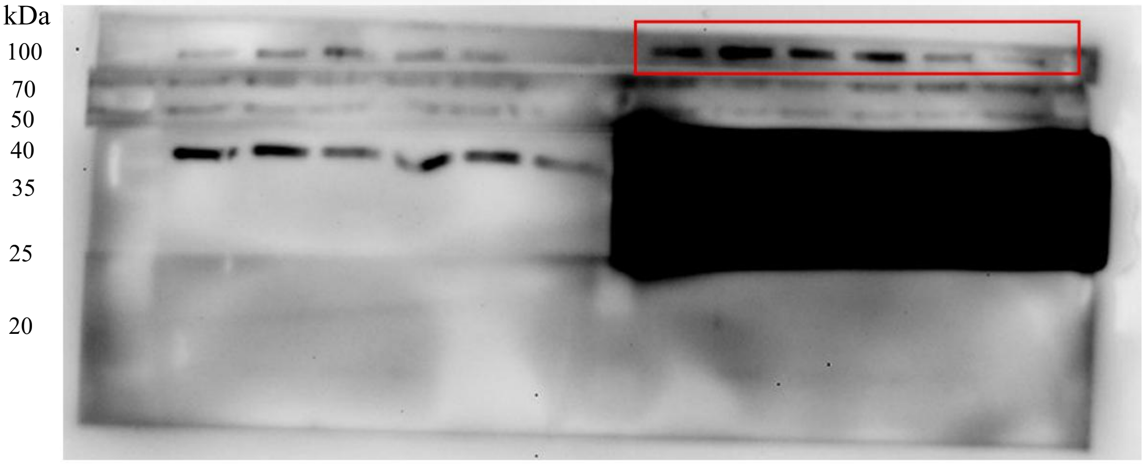

Fig5 COX-2

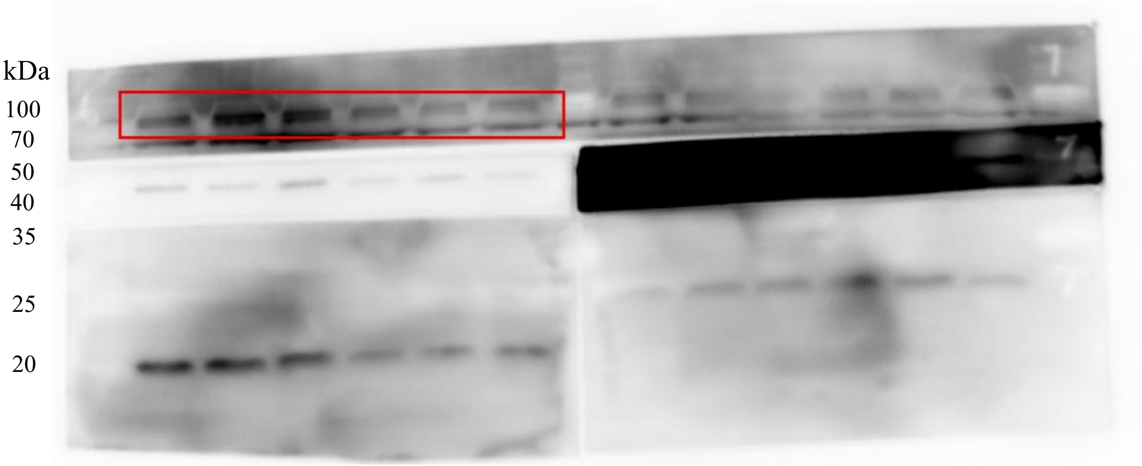

Supplement: Supplementary file 1 [file vetsci-13-00670-s001.zip › vetsci-4386936-supplementary.pdf]
